# Toxicity of *Ulva lactuca* and green fabricated silver nanoparticles against mosquito vectors and their impact on the genomic DNA of the dengue vector *Aedes aegypti*

**DOI:** 10.1049/nbt2.12082

**Published:** 2022-03-30

**Authors:** Al Thabiani Aziz

**Affiliations:** ^1^ Department of Biology Faculty of Science University of Tabuk Tabuk Saudi Arabia

**Keywords:** *Aedes aegypti*, antimicrobial, *Culex pipiens*, RAPD, *v*ectors

## Abstract

Marine seaweeds are known to have a potential role against microbial and pesticidal activities. *Ulva lactuca,* a green macroalgae extract analysed through gas chromatography mass spectrometry reveals 31 compounds. Resistance of mosquito vectors to synthetic insecticides remains a major problem. Discovering and applying natural agents to act against disease vectors is challenging. The activities of the extract and nano‐fabricated green synthesised silver nanoparticles were checked for use against *Aedes aegypti* and *Culex pipien*s. The crude extract and synthesised silver nanoparticles exhibited a notable larvicidal effect, and very effective inhibition of pupal and adult emergence. Inhibition of adult emergence of *Ae.aegypti* was 97.7% and in *Cu.pipien*s, it was 93.3%. Our genotypic study of Deoxyribonucleic acid from treated larvae utilising random primers MA‐09, MA‐12 and MA‐26 revealed damaged nucleotide sequences when compared with the controls. The antimicrobial activity of both the extract and green synthesised nanomaterials showed prominent activity against pathogenic drug resistant bacteria. Our results contribute to further development of eco‐friendly insecticides with lower cost of preparation. This could further contribute to further research helping future generations to be free from these deadly disease‐causing vectors and pathogenic microbes.

## 1 INTRODUCTION

Mosquitoes (Diptera: Culicidae) are the major vector for most of the deadly transmitted diseases in the world [1, 2] affecting more than 700,000,000 people per year throughout the world [3]. Mosquitoes have long slender body with long legs and piercing mouthparts. *Anopheles*, *Aedes*, and *Culex* are the three most common mosquito vector genera. They are biting Dipterans that suck blood from humans and other animals while acting as carriers of several diseases. Most of these diseases, such as malaria, filariasis, dengue, chikungunya, yellow fever, Zika virus, etc., are commonly spread throughout the human populations [4]. The resurgences of these diseases are attributed to multiple breeding sites in addition to resistance among mosquito populations due to repeated usage of chemical insecticides increasing resistance among mosquito population as well as causing serious environmental pollution [5]. Thus, an alternative approach is needed to fight against mosquito populations.

Seaweeds have wide range of biological applications from nutrition to therapeutic applications in pharmaceutical industries, etc. *Ulva lactuca* is a macroalgae and an edible seaweed found abundantly in the marine environment. This species is found practically everywhere in shallow waters, including estuaries, throughout the World Ocean [6, 7]. It has wide acceptance in society because it is biodegradable, eco‐friendly and cost effective. Reports have also suggested that seaweeds have potential insecticidal activity [8, 9]. Study of the bioactive compounds present in seaweed can contribute to our knowledge of their mode of action in combating mosquito larvae [9]. *U. lactuca* has long been used for the treatment of diseases in Chinese medicines [10] and is also a rich source of ash, proteins, lipids, fibre and carbohydrates [11].

*Aedes aegypti* Linn. (Diptera: Culicidae) is the major vector of dengue, haemorrhagic fever, yellow fever and chikungunya [12]. Two thirds of the world’s population harbours this dengue vector [13], including a total of more than 128 countries [14]. *Culex pipiens* L. is a commonly occurring mosquito in urban and semi‐urban areas. It acts as a primary enzootic vector of West Nile in birds and acts as the vector for transmission of this virus to humans [15]. It was found to be highly resistant against most chemical insecticides such as temephos and fenthion [16].

In addition to our direct use of botanical extracts as insecticides, green biosynthesis of nanomaterials plays a vital role for increasing effectiveness [17]. As a plant‐based synthesis, it is eco‐friendly and does not contain or generate any hazardous substances [18]. The phytoconstituents present in these natural insecticides were proven to have mosquitocidal and insecticidal properties [19]. As Ag has long been used widely for biomedical and veterinary purposes, it has demonstrated no harmful effects against humans or the environment.

In this research, an ecofriendly nano‐based insecticide was synthesised from *Ulva lactuca*. The bio‐efficacies of both the *U. lactuca* extract and green nano‐synthesised silver nanoparticles were studied against *Ae. aegypti* and *Cu. pipiens* mosquito vectors. Measures of larvicidal, pupal emergence, adult emergence and extent of inhibition were conducted. Genotoxicity of green synthesised nanoparticles on Deoxyribonucleic acid (DNA) was determined by random amplified polymorphic DNA (RAPD) analysis through agarose gel electrophoresis. Antimicrobial activity the of *U. lactuca* extract and fabricated AgNPs toxicity against pathogenic strains were studied and their efficacy was analysed.

## 2 MATERIALS AND METHODS

### 2.1 Seaweed collection and extraction

The seaweed *Ulva lactuca* was collected from the Red Sea coast of Haql (29°20′25.4″N, 34°56′53.51″E), Tabuk region, Saudi Arabia, in the early mornings. They were cleaned with distilled water, shade dried at an average temperature of 27°C and 77% relative humidity and powdered. About 10 gm of the powdered sample was aqueous extracted using a soxhlet apparatus using ethanol at a boiling point of 78°C for 8 h and concentrated with a rotary vacuum evaporator at 40°C. The yield extracted was stored at 4°C for further assays.

### 2.2 Gas chromatography mass spectrometry analysis of U. lactuca extract

The aqueous extract of *U. lactuca* was subjected to gas chromatography mass spectrometry (GC‐MS) analysis. One ml of the sample was dissolved in high‐performance liquid chromatography grade methanol and an active fraction was subjected to GC and MS JEOL GC mate equipped with a secondary electron multiplier. JEOL GCMATE II GC‐MS, an Agilent Technologies 6890N Network GC system for gas chromatography, was used. The column (HP5) contained fused silica 50 × 0.25 mm I.D and the run time was 20 min. Column temperature was programed at 110°C with a 10°C/min rise to 230°C, and 250°C was chosen for for injector temperature. Helium (99.99%) was the carrier gas, and the split ratio was 5:4. The sample (1 μL) was evaporated in a splitless injector at 280°C. The compounds were identified by gas chromatography coupled with mass spectrometry with source at 270°C and an electron impact mode at 70 eV. The molecular weight, molecular formula, and structure of the compounds of the tested materials were ascertained by interpretation on the mass spectrum of GC‐MS using the database of the National Institute Standard and Technology.

### 2.3 Synthesis and characterisation of U. lactuca‐fabricated Ag nanoparticles

About 100 ml of the *Ulva lactuca* extract was mixed with freshly prepared 1 mM AgNO_3_ solution. A yellowish colour indicated the formation of *U.lactuca‐*fabricated AgNPs. The synthesised green based AgNPs were further characterised by Uv‐Vis, transmission electron microscopy (TEM), energy‐dispersive X‐ray spectroscopy (EDAX), Zeta potential and X‐ray diffraction (XRD) (Phillips PW1830, 40 kV, 30 mA with CuKα radiation).

### 2.4 Mosquito culture

Saudi Arabian strains of *Ae.aegypti* and *Cu. pipiens* mosquitoes were cultured in an entomology laboratory at Tabuk University, Saudi Arabia, for more than 6 years without exposure to insecticides. Vectors were maintained at 27 ± 2^◦^C and 75%–85% relative humidity under a 14:10 L/D photoperiod. Brewer's yeast and dog biscuits were used as diet for larvae.

Pupae obtained from the culture were moved to a small container with tap water and placed inside the screened cages of (50 × 50 × 50 cm) for emergence of adults. The adults that emerged were deprived of sugar for 12 hours and housed with a mouse placed in resting cages overnight for blood feeding by females on the fifth day. Adults were maintained and the larvae that emerged from the eggs were used for our experiments.

### 2.5 Larvicidal bioassays

An aqueous extract was prepared, and 1 gm of crude extract was dissolved in 100 ml of distilled water. Then it was used for different ppms. Bioassays were performed for fourth instars of *Ae. aegypti* and *Cu. pipiens* at different concentrations of the crude extract, with *U. lactuca* at 100, 300, 500, 700 and 900 ppm and 50, 100, 150, 200 and 250 ppm of *U. lactuca* fabricated AgNPs.

### 2.6 Developmental studies

The larvae treated with different concentrations of the extract and *U.lactuca* fabricated AgNPs were observed for any developmental changes in their further stages. The larvae were observed, and measurements were taken. Rates of pupation, adult emergence and inhibition of emergence were calculated.

The mortality rate observed was noted after 24 h and the Inhibitory concentration (IC_50_ and IC_90_) was calculated by using Probit analysis (Minitab^®^17). The experiment was repeated for five replicates and the percentage of mortality was calculated by using Abbott's [20] formula.

### 2.7 DNA extraction from *Ae. aegypti*

DNA was extracted from *Ae. aegypti* larvae through an extraction kit obtained from DNeasy Blood & Tissue Kits (Qiagen). The larval tissue was chopped, and 10 mg of tissue placed in 1.5 ml centrifuge tubes to which we added 180 µL of ATL buffer. To this was added 20 μL of proteinase K. This was then incubated at 56°C in a shaking water bath until the tissue was lysed. The mixture was vortexed for 15 s, adding 200 μL of AL buffer to the sample. After vortexing, we added 200 μL of ethanol (96%–100%). This mixture was transferred to aDNeasy Mini spin column and centrifuged at ≥6000 x g (8000 rpm) for 1 min. We then discarded the flow through and added 500 μL AW1buffer and centrifuged for 1 min at ≥6000 x g (8000 rpm). Again the flow through was discarded, and we added 500 μL Buffer AW2 and centrifuged for 3 min at 20,000 x g (14,000 rpm). We placed the column in new centrifuge tube and added 200 μL of AE buffer, incubated it at room temperature for 1 min and then centrifuged for 1 min at ≥ 6000 x g (8000 rpm). The obtained elute contained the concentrated DNA.

### 2.8 Randomly amplified polymorphic DNA‐PCR

The genomic DNA extracted from *Ae. aegypti* was amplified using random primers for further studies. The Polymerase Chain Reaction (PCR) mixtures were prepared with a final volume of 15 μL, the master mix containing 1x PCR buffer 1.5 μL, MgCl2 (1 mM) 0.6 μL, dNTP (0.2 mM) 0.3 μL, bovine serum albumin (0.53 μL/ml) 0.8 μL, primers (MA‐09, MA‐12 and MA‐26) 0.3 μL and Taq (0.3 μL). About 1 μL of the DNA elute was added to the master mix and the final volume was made with deionised water. DNA primers added were MA‐09 GACGGATCAG, MA‐12 ACCGCGAAGG, MA‐26 GACGTGGTGA which have annealing temperatures of 32°C, 34°C and 32°C, respectively. The final mixture was loaded into a PCR machine for further amplification through denaturation, 94°C for 1 min, annealing temperature of 32°C, extension at 72°C for 2 min and a final step of 72°C for 10 min.

### 2.9 Agarose gel electrophoresis

The amplified DNA fragments of *Ae. aegypti* through PCR were analysed by agarose gel electrophoresis based on the procedure followed by Lalli et al. [21]. Agarose gel was prepared with 1.5% Tris‐acetate‐EDTA (TAE) buffer in which amplified DNA fragments through RAPD were mixed with bromophenol blue dye at a 5: 1 ratio. This was loaded carefully into the electrophoresis chamber wells along with a standard DNA marker containing TAE buffer (4.84 g Tris base, pH 8.0; 0.5 M EDTA/1 L) and finally loaded onto the agarose gel (1.5% gel stained with 10 μg/ml of ethidium bromide). The gel containing samples was connected to the power supply (120 V) for 45 min and the DNA bands were observed under the UV transilluminator to determine the profiles of the treated insects.

### 2.10 Antibacterial activity

The antibacterial activities of *U. lactuca* and the fabricated AgNPs were determined by using the agar disc diffusion method on Muller Hinton agar medium. The bacterial strains were cultured initially in a nutrient broth prior to use. Test organisms used were *Klebsiella pneumoniae* (KP) American Type Culture Collection (ATCC) *13,883, Pseudomonas aeruginosa* (PA) *ATCC 27,853*, *Staphylococcus aureus* (SAU) ATCC 25,923, and *Methicillin‐resistant Staphylococcus aureus* (MRSA) ATCC 43,300. An antibiotic disk Neomycin 30 μg (Neo30) was used as a positive control and sterile distilled water was used as a negative control. The inhibition of the extract was calculated by their zone of inhibition. The study organism was obtained from the Department of Biology, University of Tabuk, Tabuk, Kingdom of Saudi Arabia.

### 2.11 Statistical analysis

The mortality numbers obtained from our experiment were subjected to analysis of variance (ANOVA of arcsine, logarithmic and square root transformed percentages). Significant differences between treated and control groups were analysed by Tukey's multiple range test (significance at *p* < 0.05) using the Minitab^®^17 programme. Probit analysis with a dependability interval of 95% using the Minitab^®^17 programme was used to find the lethal concentrations required to kill 50% (LC_50_) of larvae in 24 h.

## 3 RESULTS

**FIGURE 1:**
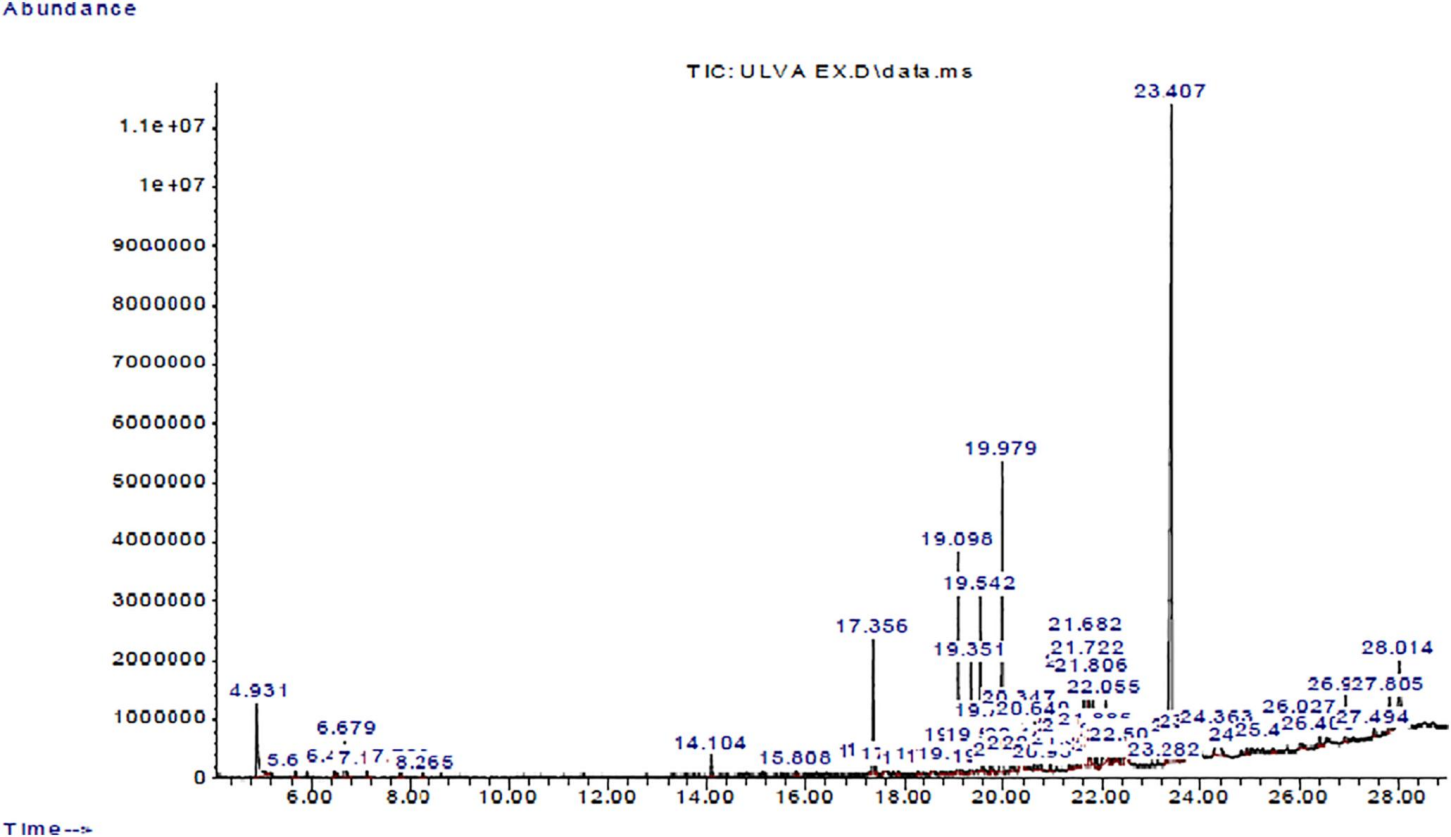
GC‐MS chromatogram of *Ulva lactuca* aqueous extract with reference to time and abundance

**FIGURE 2:**
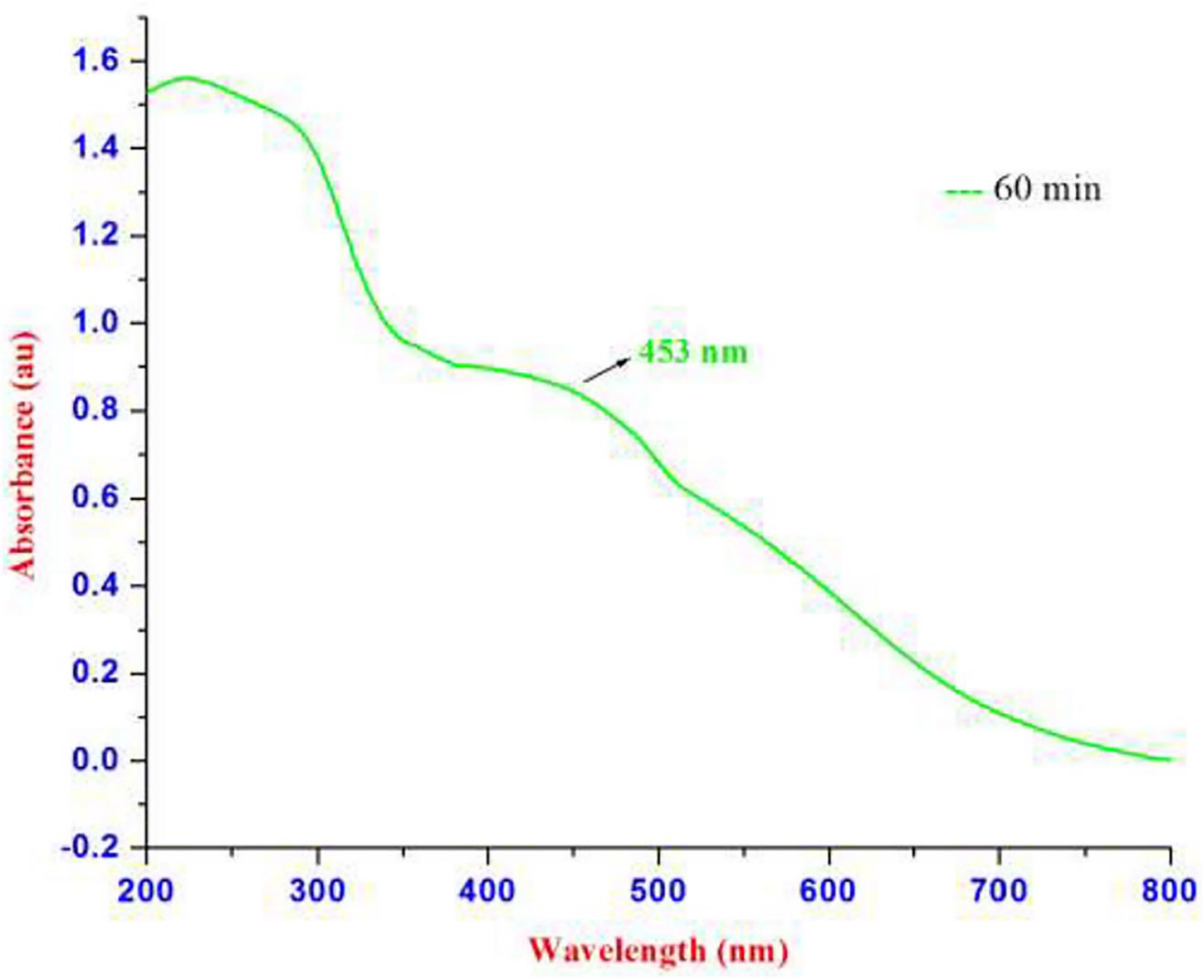
UV‐Vis spectrum of AgNPs synthesised by the *Ulva lactuca* extract

**FIGURE 3:**
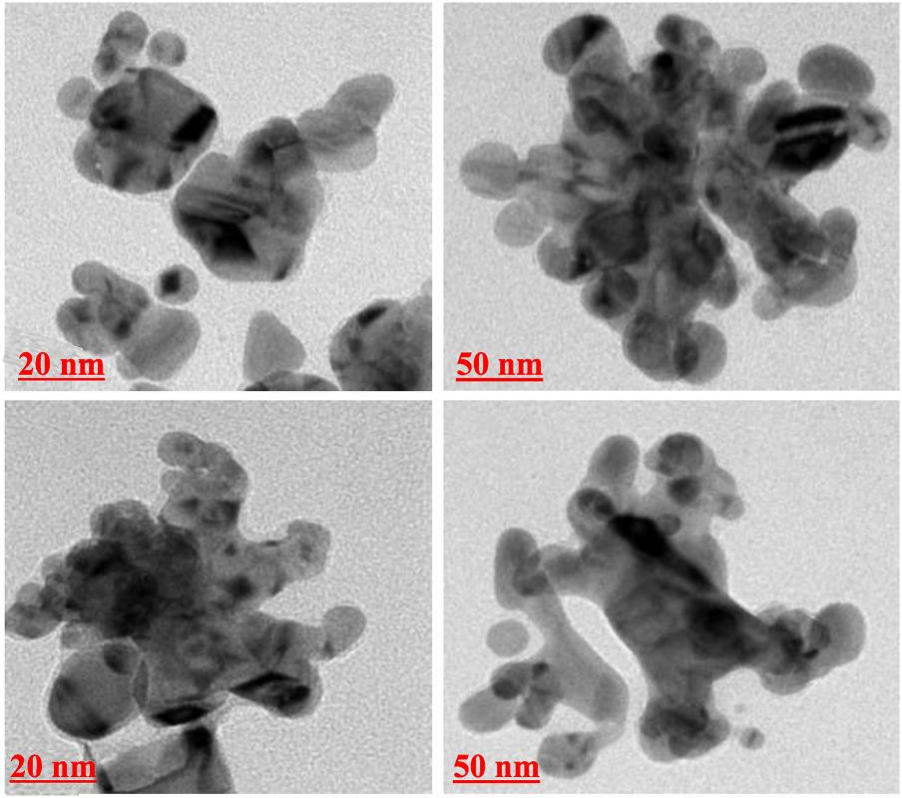
TEM of AgNPs synthesised using the *U. lactuca* extract

**FIGURE 4:**
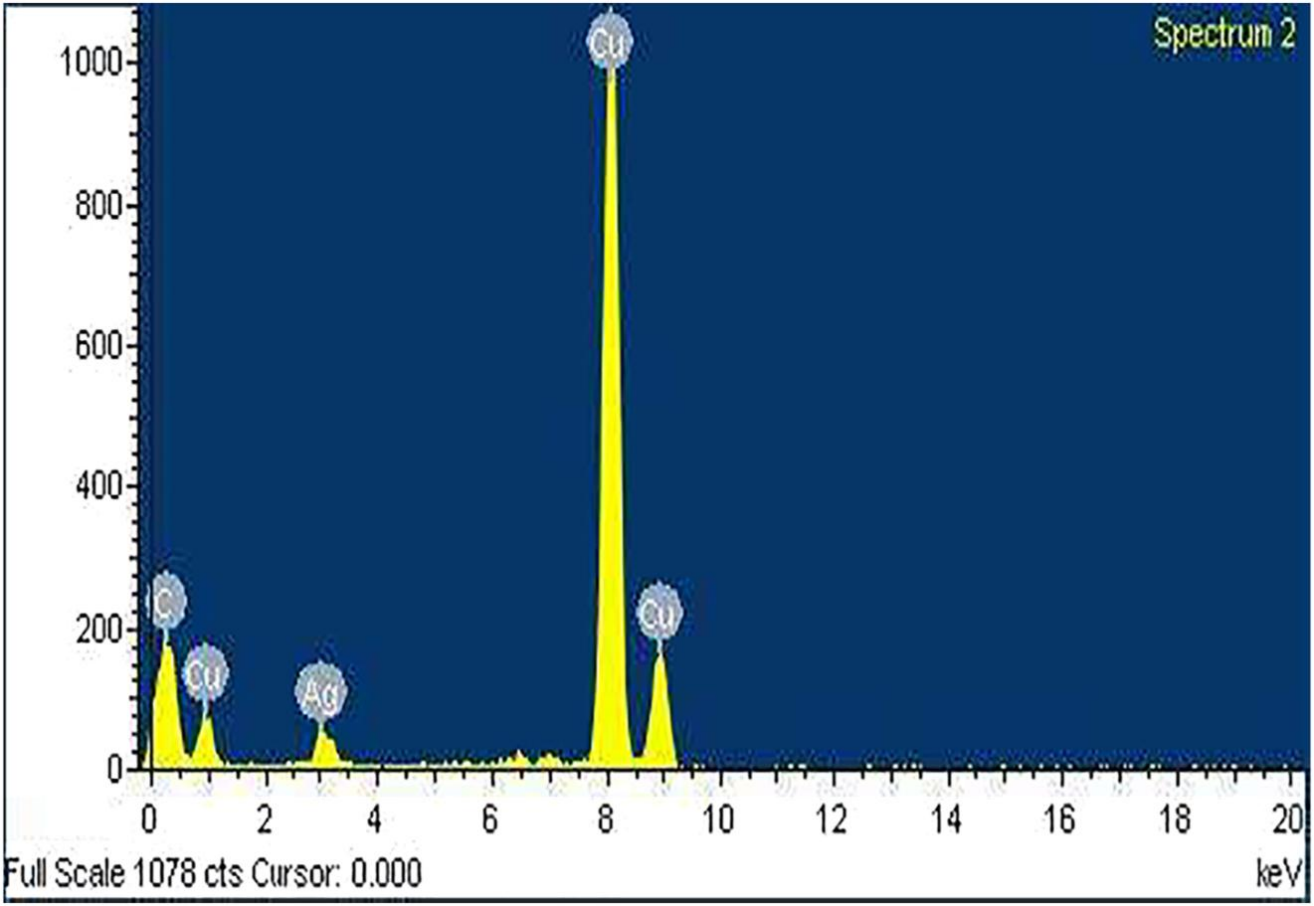
EDX spectroscopy of AgNPs synthesised using the *Ulva lactuca* extract

**FIGURE 5:**
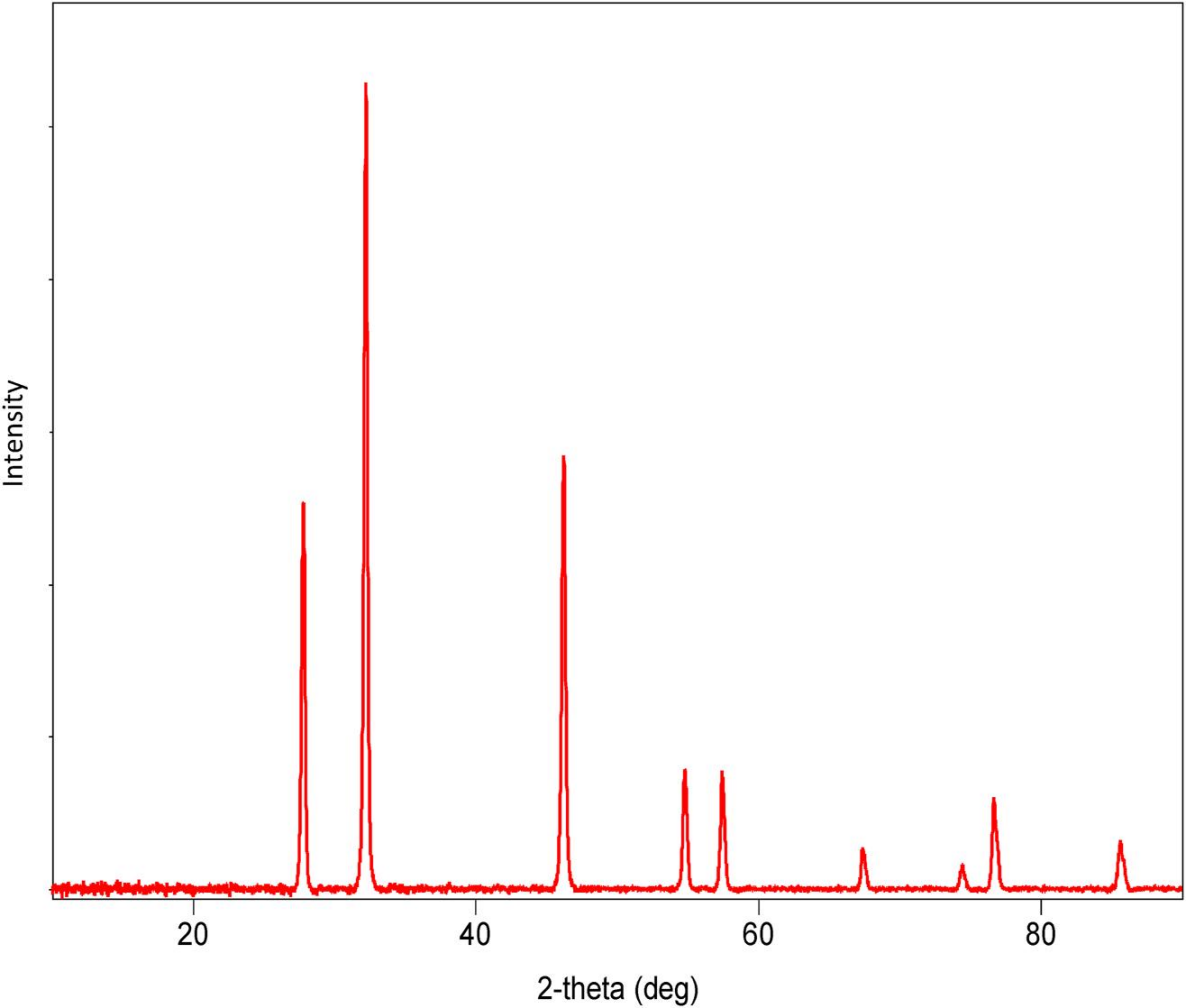
XRD of *Ulva lactuca* fabricated AgNPs

**FIGURE 6:**
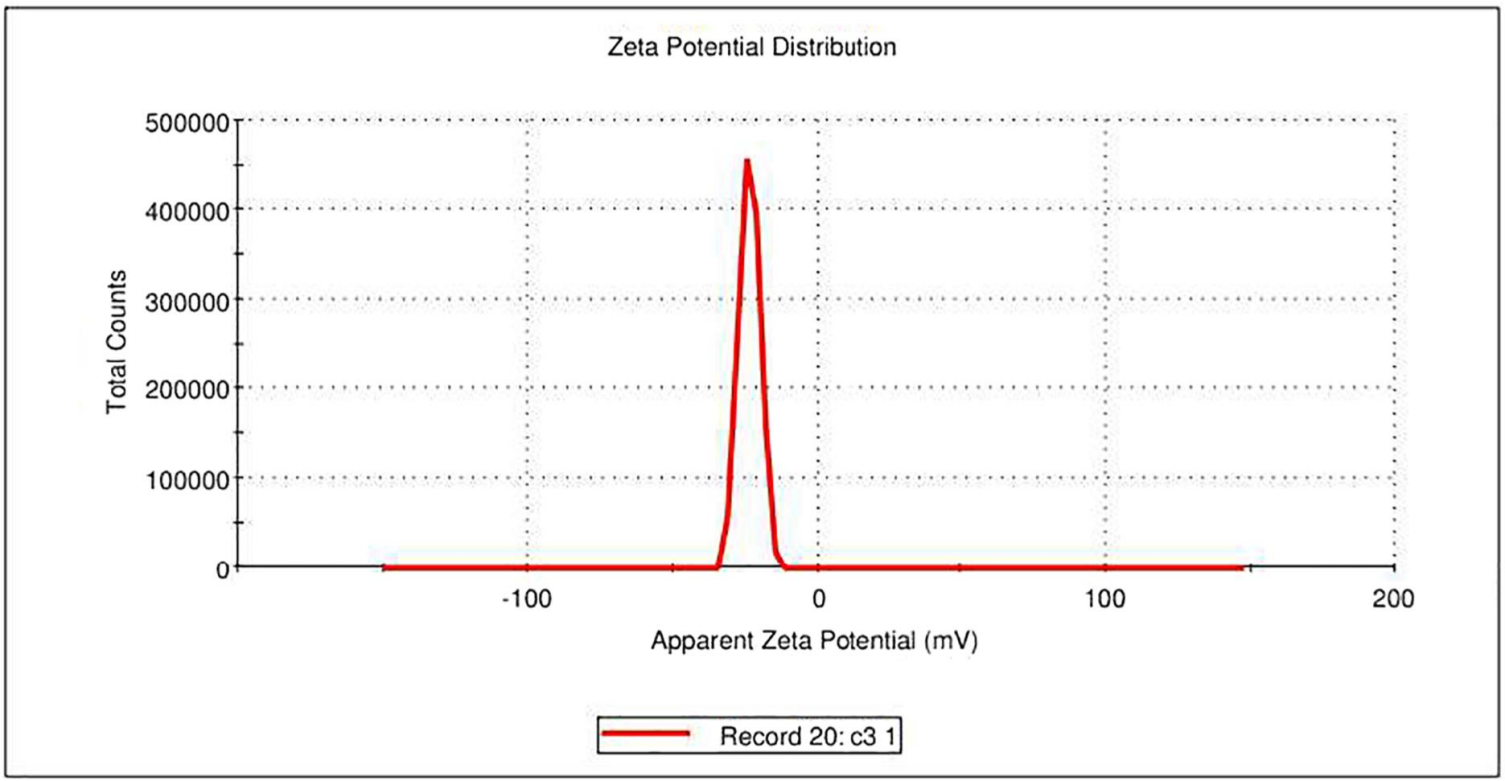
Zeta potential of *Ulva lactuca* fabricated AgNPs

The results obtained from the GC‐MS analysis of aqueous extracted *U. lactuca* show the presence of 31 compounds as shown in Figure 1 and the compounds were Heptanal, 2‐Furancarboxaldehyde, 5‐methyl, 2(3H)‐Furanone, 5‐ethyldihydro, 10‐Undecenoic acid, methyl ester 2(4H)‐Benzofuranone, 5,6,7,7a‐tetrahydro‐4,4,7a‐trimethyl‐, 8‐Heptadecene 3‐Buten‐2‐one, 4‐(4‐hydroxy‐2,2,6‐trimethyl‐7‐, Oxabicyclo [4.1.0]hept‐1‐yl)‐, Methyl tetradecanoate, Tetradecanoic acid, Pentadecanoic acid, methyl ester, 2‐Cyclohexen‐1‐one, 4‐hydroxy‐3,5,5‐trimethyl‐4‐(3‐oxo‐1‐butenyl)‐, Methyl 13‐methyltetradecanoate, 2‐Hexadecene, 3,7,11,15‐tetramethyl‐, [R‐[R*,R*‐(E)]]‐, Bicyclo [3.1.1] heptane, 2,6,6‐trimethyl‐, 2‐Hexadecene, 2,6,10,14‐tetramethyl‐, 9‐Octadecyne, 3,7,11,15‐Tetramethyl‐2‐hexadecen‐1‐ol, Methyl 4,7,10,13‐hexadecatetraenoate, 9‐Hexadecenoic acid, methyl ester, (Z)‐, Hexadecanoic acid, methyl ester, Z‐7‐Hexadecenoic acid, n‐Hexadecanoic acid, Pentanoic acid 1‐methylpropyl ester, Ethyl 9‐hexadecenoate, Hexadecanoic acid, ethyl ester, *cis*‐9‐Hexadecenoic acid, Methyl 10‐methyl‐hexadecanoate, gamma. Dodecalactone, 13‐Hexyloxacyclotridec‐10‐en‐2‐one, Methyl stearidonate and 9,12‐Octadecadienoic acid (Z, Z)‐, methyl ester were detected and varied in their retention time with absorption peaks etc (Table 1). The aqueous extract of *U. lactuca* incubated with AgNO_3_ showed a yellowish colour which indicates the formation of *U. lactuca‐*fabricated AgNPs which was confirmed by UV‐vis spectroscopy (Figure 2) and the absorption maxima of *U. lactuca‐*synthesised AgNPs was at 453 nm. The particle size of the synthesised particles ranging 20‐50nm is shown in Figure 3. The elemental composition of *U. lactuca*‐synthesised AgNPs show copper and carbon by EDAX spectroscopy in Figure 4. The XRD profile of *U. lactuca‐*fabricated AgNPs shows prominent peaks. The characteristic study of green synthesised *U. lactuca‐*fabricated AgNPs shows a definitive absorption peak at near 8 keV, which is shown in Figure 5. The zeta potential values of *U. lactuca‐*synthesised AgNPs were found to have a negative zeta potential and confirm the stability developed formulation (Figure 6).

**TABLE 1:** Compounds identified in *U. lactuca* using gas chromatography mass spectrometry (GC‐MS)

S. No	Retention time (min)	Molecular formula	Compound name	Molecular weight	Absorbance area	Structure
1	5.67	C_7_H_14_O	Heptanal	114.104	186,390	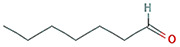
2	6.46	C_6_H_6_O	2‐Furancarboxaldehyde, 5‐methyl‐	110.037	248,588	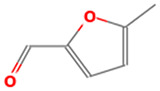
3	8.26	C_6_H_10_O_2_	2(3H)‐Furanone, 5‐ethyldihydro‐	114.068	203,977	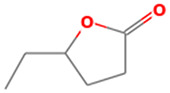
4	14.10	C_12_H_22_O_2_	10‐Undecenoic acid, methyl ester 2(4H)‐Benzofuranone, 5,6,7,7a‐tetrahydro‐4,4,7a‐trimethyl‐,	198.162	617,988	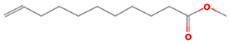
5	15.8	C_11_H_16_O_2_	2‐Isopropoxy‐1‐phenylethanol 1‐Phenyl‐2‐isopropoxyethanol	180.115	198,812	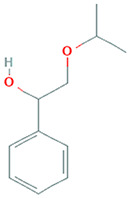
						
6	17.35	C_9_H_10_N_4_O_2_S	8‐Heptadecene 3‐Buten‐2‐one, 4‐(4‐hydroxy‐2,2,6‐trimethyl‐7‐	238.266	3,618,496	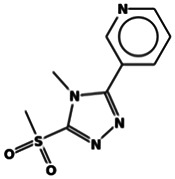
7	17.57	C_13_H_20_O_3_	Oxabicyclo [4.1.0]hept‐1‐yl)‐	224.141	285,607	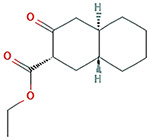
8	17.86	C_15_H_30_O_2_	Methyl tetradecanoate	242.225	237,377	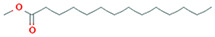
9	18.27	C_14_H_27_O_2_^−^	Tetradecanoic acid	228.209	217,674	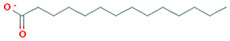
10	18.55	C_16_H_32_O_2_	Pentadecanoic acid, methyl ester	256.24	192,444	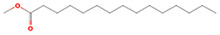
11	18.77	C_14_H_20_O_3_	2‐Cyclohexen‐1‐one, 4‐hydroxy‐3,5,5‐trimethyl‐4‐(3‐oxo‐1‐butenyl)‐	222.126	264,781	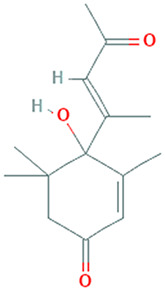
12	18.94	C_16_H_32_O_2_	Methyl 13‐methyltetradecanoate	256.24	204,863	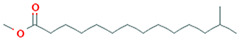
13	19.03	C_20_H_40_	2‐Hexadecene, 3,7,11,15‐tetramethyl‐, [R‐[R*,R*‐(E)]]‐	280.313	152,101	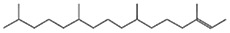
14	19.09	C_10_H_18_	Bicyclo [3.1.1]heptane, 2,6,6‐trimethyl‐	138.14	5,964,059	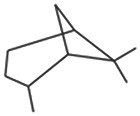
15	19.16	C_20_H_40_	2‐Hexadecene, 2,6,10,14‐tetramethyl‐	280.313	662,693	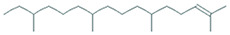
16	19.35	C_18_H_34_	9‐Octadecyne	250.266	2,780,276	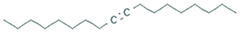
17	19.54	C_20_H_40_O	3,7,11,15‐Tetramethyl‐2‐hexadecen‐1‐ol	296.308	4,615,395	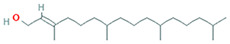
18	19.59	C_17_H_26_O_2_	Methyl 4,7,10,13‐hexadecatetraenoate	262.193	563,615	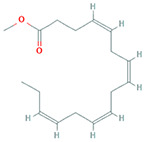
19	19.73	C_17_H_32_O_2_	9‐Hexadecenoic acid, methyl ester, (Z)‐	268.24	150,655	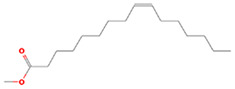
20	19.97	C_17_H_34_O_2_	Hexadecanoic acid, methyl ester	270.256	8,872,106	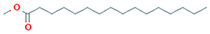
21	20.17	C_16_H_30_O_2_	Z‐7‐Hexadecenoic acid	254.225	469,538	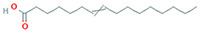
22	20.34	C_16_H_32_O_2_	n‐Hexadecanoic acid	256.24	1,869,464	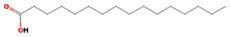
23	20.40	C_9_H_18_O_2_	Pentanoic acid 1‐methylpropyl ester	158.131	280,366	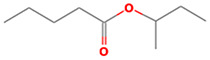
24	20.44	C_18_H_34_O_2_	Ethyl 9‐hexadecenoate	282.256	272,685	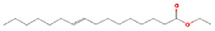
25	20.64	C_18_H_36_O_2_	Hexadecanoic acid, ethyl ester	284.272	1,414,426	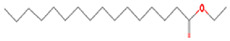
26	20.755	C_16_H_30_O_2_	*cis*‐9‐Hexadecenoic acid	254.255	489,299	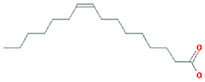
27	20.95	C_18_H_36_O_2_	Methyl 10‐methyl‐hexadecanoate	284.272	190,180	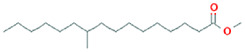
28	21.33	C_12_H_22_O_2_	Gamma. Dodecalactone	198.162	477,903	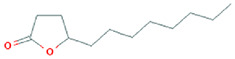
29	21.44	C_18_H_32_O_2_	13‐Hexyloxacyclotridec‐10‐en‐2‐one	280.24	896,867	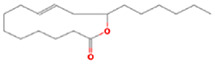
30	21.54	C_19_H_30_O_2_	Methyl stearidonate	290.225	851,195	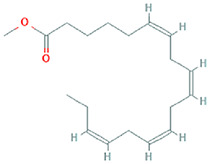
31	21.61	C_19_H_34_O_2_	9,12‐Octadecadienoic acid (Z,Z)‐, methyl ester	294.256	2,107,321	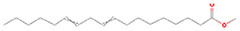

**FIGURE 7:**
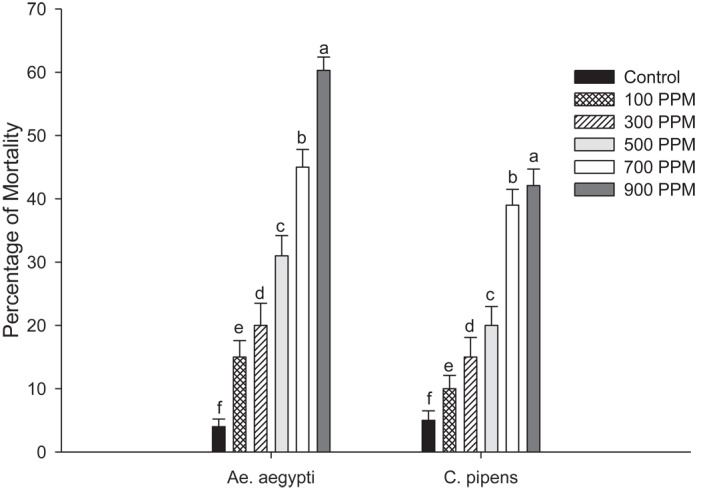
Larvicidal activity of *U. lactuca* extract against mosquito strains of *Ae. Aegypti* and *Cu. pipiens*

**FIGURE 8:**
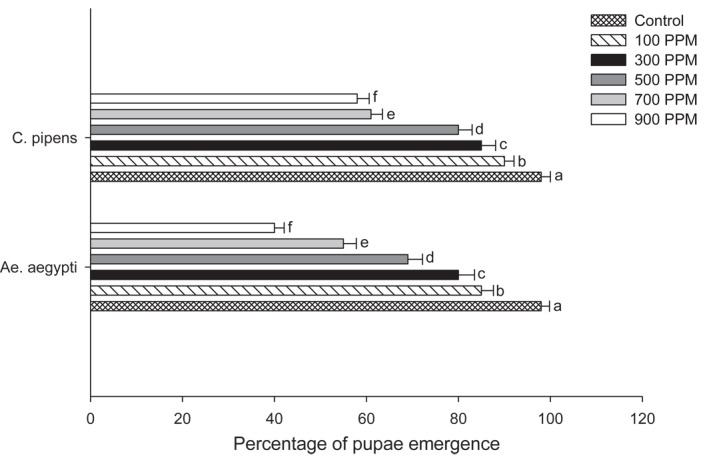
Emergence of pupae of two mosquito strains after treatment with *U. lactuca* extract

**FIGURE 9:**
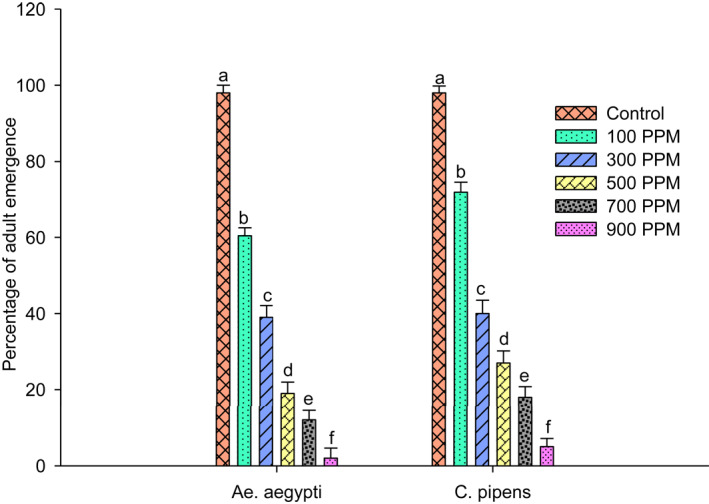
Emergence of adults of two mosquito strains after treatment with *U. lactuca* extract

**FIGURE 10:**
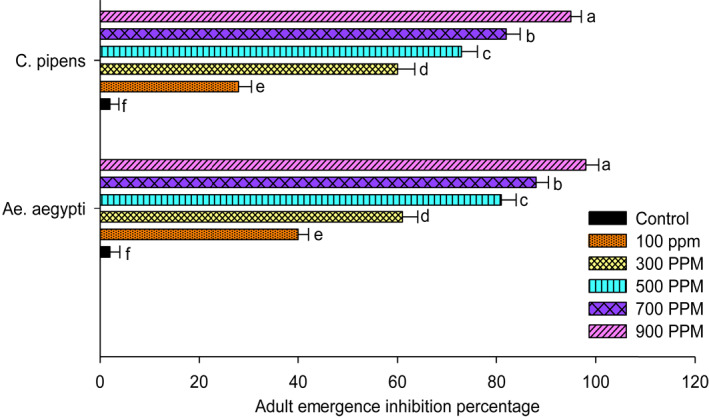
Inhibition of adult emergence of two mosquito strains after treatment with *U. lactuca* extract

The *U. lactuca* extract against strains of *Ae. aegypti* and *Cu.pipiens* showed variation in their activity when exposed at 100, 300, 500, 700 and 900 ppm. The larvae show a dose‐dependent mortality with significant differences in their results. The highest mortality of 60.3% was noted in *Ae. aegypti* with 42.1% in *Cu. pipiens* at 900 ppm concentration. The pupae emergence from the treated larvae showed higher percentage of 90% at 100 ppm concentration in *Cu. pipiens* and 85% in *Ae. aegypti*. Adult emergence was reduced, and the treated larvae showed 2.3% reduction in *Ae.aegypti* and 5.1% reduction in *Cu. pipiens* at a higher concentration of 900 ppm. Thus, the adult inhibition percentage is higher at higher concentrations; in *Ae. aegypti* it was 98% and 95% in *Cu. pipiens.* Results are shown in Figures 1, 2, 3, 4, 5, 6, 7, 8, 9, 10. The IC_50_ value of *Ae. aegypti* was 165.42 ppm and for *Cu. pipiens* was 220.27 ppm. For *U. lactuca* extract, the IC_95_ value for *Ae. aegypti* was 758.76 ppm and for *Cu.pipiens* 946.54 ppm, respectively.

**FIGURE 11:**
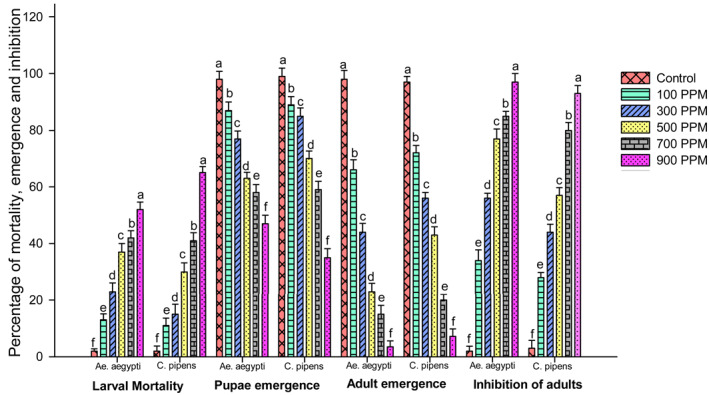
Percentage of mortality, pupae emergence, adult emergence and inhibition of adults after treatment with *Ulva lactuca* fabricated AgNPs

Larvae treated with *U.lactuca‐*fabricated AgNPs showed better activity at lower concentrations. The highest mortality of 65.5% in *Cu. pipiens* was at 250 ppm and at 52.2% in *Ae. aegypti.* The emergence of pupae from treat larvae was lowered by 35.6% in *Cu. pipiens* and 48.7% in *Ae. aegypti*. Adult emergence was also reduced through treated *U. lactuca‐*fabricated AgNPs by 3.5% in *Ae. aegypti* and 7.2% in *Cu. pipiens.* This implies an inhibition percentage of 97.7% in *Ae. aegypti* and 93.3% in *Cu. pipiens.* Results from *Ulva lactuca‐*fabricated AgNPs are shown in Figure 11. The inhibitory concentration of *U. lactuca‐*fabricated AgNPs against *Ae. aegypti* is 80.51 and 105.65 ppm for *Cu.pipiens*. The IC_95_ value against *Ae. aegypti* is 226.9 ppm and for *Cu.pipiens* is 337.19 ppm.

The impact of *U.lactuca* extract against *Ae. aegypti* larvae exposed to LC_50_ shows variation in their genomic DNA. Results from the RAPD profile of the DNA through agarose gel electrophoresis show variations in the intensity of bands. The DNA isolated from treated *Ae. aegypti* larvae amplified with MA‐09, MA‐12 and MA‐26 show modifications in their genome which was clearly evident from the control larvae. The DNA isolated from treated larvae from LC_50_ and LC_90_ showed variation in their amplification. Amplified DNA through MA‐09 showed 5 bands in control correspondence with 500 bp, 600 bp, 800 bp, 1000bp and 1500 bp. Four bands were obtained from LC_50_ larvae at 500 bp, 700 bp, 800 bp and 1500 bp and, from LC_90_ larvae 4 bands correspond with molecular sizes of 500 bp, 600 bp, 700 bp, 800 bp.

**FIGURE 12:**
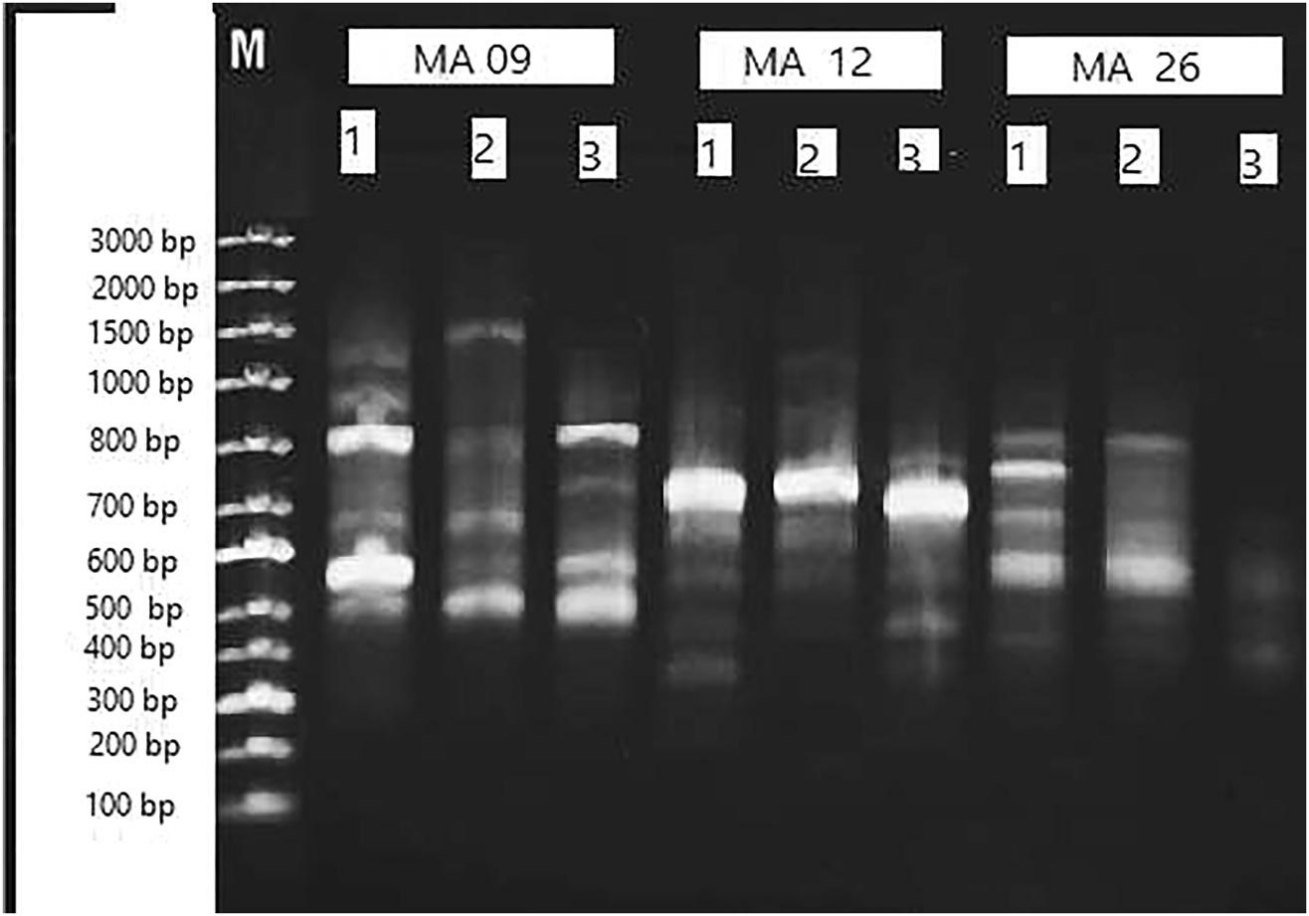
RAPD analysis of DNA isolated from *Ae. aegypti* amplified with random primers. One‐ Control, 2‐LC_50_, 3‐ LC_90_

DNA amplified from the MA‐12 marker revealed observance of 5 bands in control larvae and 4 bands from LC_50_ larvae and 3 bands from LC_90_ larvae. Amplification of DNA through MA‐26 revealed four bands in control in correspondence with 600, 700, 800 and 900 bp. In treated larval DNA from LC_50_ there were four bands observed. Only one band was observed from LC_90_‐treated larval DNA. The results obtained are shown in Figure 12.

**FIGURE 13:**
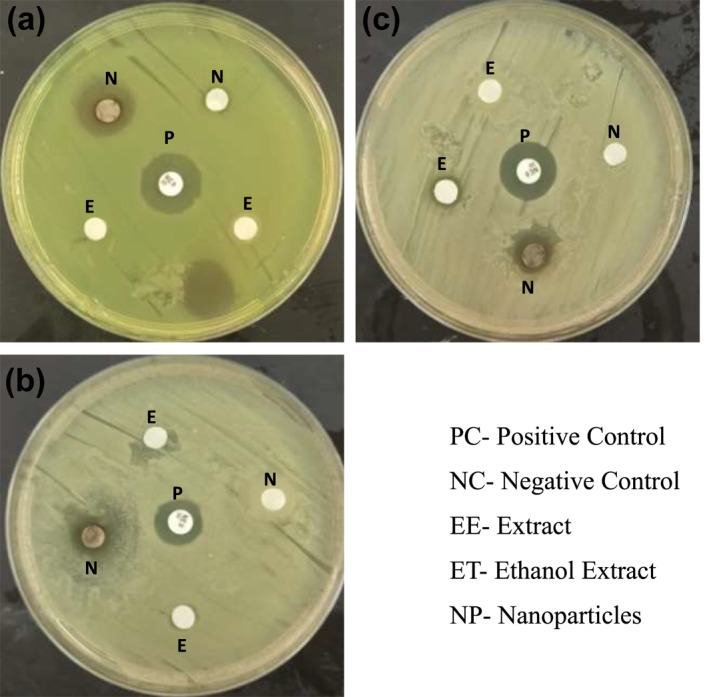
Antimicrobial activity of *Ulva lactuca* extract and its mediated AgNPs

Antimicrobial activity of *U. lactuca* extract against *KP, PA, Staphylococcus aureus* and *MRSA* show variation in their activity, which is revealed through their zones of inhibition. The inhibition of *U. lactuca* against *S*. a*ureus* displayed good activity by 9 mm, followed by MRSA*, P. aeruginosa* and *K. pneumoniae*. The activity of *U. lactuca‐*fabricated AgNPs against strains revealed considerable activity against *P. aeruginosa* followed by *K. pneumoniae > S. aureus >*the *MRSA* strain. Some activity revealed from *U. lactuca‐*fabricated AgNPs indicated better activity than the positive control. Discs diffused with ethanol and the negative control revealed no zone of inhibition. Results obtained are shown in Figure 13.

## 4 DISCUSSION

Development of eco‐friendly natural pesticides against insects has a major role in public health and environment protection. Seaweeds that can be obtained in enormous quantities and having non‐harmful properties, can be used widely for their biological activity [1]. Gas chromatography mass spectrometry analysis of aqueous extracted *U. lactuca* shows the presence of 31 compounds. Similar ethanolic extracts of *U. lactuca* revealed 17 compounds [22]. Most of the compounds identified from *U. lactuca* have medicinal properties previously identified by researchers [23]. And controlling vector population by green synthesised AgNPs is now an emerging technique [24].

The nanoparticle synthesised from *U. lactuca* extract is evident by the colour changes; this may likely be due to surface plasmon resonance oscillation of the nanoparticles. An absorption peak for seaweed‐fabricated AgNPs is noticed at 453 nm. Comparably, *Sargassum muticum‐*synthesised AgNPs show a maximum absorption peak at 400 nm [25]. TEM analysis reveals a particle size of 20–50 nm for this synthesised nanoparticle. Earlier researchers [25, 26, 27] consider particles of 1–100 nm and solid particles of 10–1000 nm [24] to be nanoparticles.

The *U. lactuca‐*fabricated AgNPs show positive anti‐vector results when compared with crude extract. This was evident from the observed vector mortality percentages of treated strains. This is due to lower particle size and easier penetration of target insect larvae, thus providing more advantages than conventional insecticides. The plant‐mediated and fabricated nanoparticles are synthesised in a single‐step; therefore, there is no requirement of high pressure, high temperature or more toxic chemicals, etc. [28].

The toxicity of *U. lactuca* crude extract towards strains shows highest mortality against *Ae. aegypti* when compared with *Cu. pipiens.* This suggests *Ae. aegypti* are more susceptible to the extract. Pupal emergence and adult emergence from the treated larvae were also observed to be lower. Inhibition percentage of this strain was greatest at *Ae. aegypti* (98%) when compared with *Cu. pipiens.* Similar to our research, Suresh et al. [29] observed the aqueous leaf extract of *S. maritima* against *Ae. aegypti*. The active compounds which were present in the green extracts were more active against disease vectors, binding to target sites and providing better results. This suggests that aqueous extracts are toxic towards larvae in the same way *Gracilaria firma* extracts are toxic against *Ae. aegypti* at an earlier stage [30].

An inhibitory concentration of *U. lactuca* against *Ae. aegypti* was obtained at a lower concentration when compared with *Cu. pipiens.* Fabricated AgNPs show their highest mortality in *Cu.pipiens* (65.5%) and pupal emergence was also lower in *Cu.pipiens,* but adult emergence was lower in *Ae. aegypti* when compared with *Cu. pipiens.* This implies a decrease in reduction of adult emergence which contributes towards an environment free from *Ae. aegypti.* Even though our extract showed reduced mortality against *Ae. aegypti,* adult emergence and emergence inhibition was observed to be higher. Studies focussed on plant fabricated nanoparticles against mosquito larvae are highly toxic at low concentrations [31].

The green‐fabricated AgNPs showed the highest percentage of mortality at the minimum concentration of 250 ppm in *Cu. pipiens* when compared with *Ae. aegypti.* Thus, a small quantity of the synthesised nanoparticle has a capability to bind to the insect cuticle, penetrate inside the cells, and destroy further functions of the cells by protein synthesis, enzymatic activity, etc. *Artemisia*‐synthesised AgNPs exhibited similar results when exposed to mosquito vectors [32].

The RAPD‐PCR analysis of treated larvae revealed a higher number of bands in the control larvae when compared with results obtained from LC_50_ and LC_90_ larvae. This clearly indicates that the random primers were unable to find their complementary sequences for amplification. The larvae treated by synthesised AgNPs might have denatured the DNA of the larvae and this could delete the base pairs, or cause nversion, mutation, or other DNA damages so that the DNA was not able to recognise random primers to amplify. In any case, the DNA was damaged by the treatment concentrations in the larvae. Similar study made on microbes from the extract of *Capparis spinosa* showed a genotoxic effect which was confirmed through RAPD‐PCR [33].

Antimicrobial activity of the *U. lactuca* extract and synthesised AgNPs shows potential against pathogenic microbes such as *KP, PA, Staphylococcus aureus* and *MRSA.* Our study results revealed synthesised that nanoparticles from *U. lactuca* show good activity measured by their zone of inhibition by the disc diffusion method, in comparison with crude extract of *U. lactuca.* Similarly antimicrobial activity of green‐synthesised AgNPs against gram positive *Staphylococcus aureus* and gram‐negative *Escherichia coli, PA* and *Enterobacter* spp. tthrough the disc diffusion method implies good activity [34]. This implies that green‐synthesised nanoparticles are more active against microbial strains than are crude extracts. It is evident from earlier research that AgNPs‐synthesised nanoparticles from *Areca catechu* exhibited potential activity against antibiotic‐resistant bacteria [35]. The compound phlorotannin from brown seaweeds also exhibits good antiviral, antibacterial, antifungal, and larvicidal activities [36].

Thus, seaweeds possess good insecticidal properties, and this activity increases when fabricated to form AgNPs. Researchers found seaweeds are more active against insects than terrestrial plant‐derived bio‐insecticides [8]. These synthesised AgNPs have a potential to control *Ae. aegypti* and *Cu*. *pipiens,*thus acting as a promising tool to control and eradicate vector borne diseases.

## 5 CONCLUSION

Insecticides of chemical origin are highly toxic towards pests but often persist inside the environment and remain toxic for future generations. This has adverse effects on biological systems and creates abnormal ecological changes. Plant‐based fabricated nanoparticles are an eco‐friendly intervention to address some of the present problems with chemical pesticides. AgNP‐based nano‐formulations are proven to be effective in our study compared to aqueous extracts. This research contributes towards possible use of herbal nanopesticides against certain deadly vector‐borne diseases. Further research needs to focus on the mode of action, stability, toxicity and non‐target effects of these agents against beneficial insects.

## CONFLICT OF INTEREST

All the authors declare no conflict of interest.

## Data Availability

The data used to support the research findings are included within the manuscript.
